# Label-free correlative morpho-chemical tomography of 3D kidney mesangial cells

**DOI:** 10.1117/1.JBO.31.3.036501

**Published:** 2026-03-10

**Authors:** Ankit Butola, Biswajoy Ghosh, Jaena Park, Minsung Kwon, Alejandro De la Cadena, Sudipta S Mukherjee, Rohit Bhargava, Stephen A. Boppart, Krishna Agarwal

**Affiliations:** a UiT the Arctic University of Norway, Department of Automation and Process Engineering, Tromsø, Norway; b UiT The Arctic University of Norway, Department of Physics and Technology, Tromsø, Norway; c University of Illinois Urbana-Champaign, NIH/NIBIB P41 Center for Label-free Imaging and Multiscale Biophotonics, Urbana, Illinois, United States; d University of Illinois Urbana-Champaign, Beckman Institute for Advanced Science and Technology, Urbana, Illinois, United States; e University of Illinois Urbana-Champaign, Department of Bioengineering, Urbana, Illinois, United States; f University of Illinois Urbana-Champaign, Department of Chemical and Biomolecular Engineering, Urbana, Illinois, United States; g University of Illinois Urbana-Champaign, Department of Electrical and Computer Engineering, Urbana, Illinois, United States; h University of Illinois Urbana-Champaign, Cancer Center at Illinois, Urbana, Illinois, United States; i University of Illinois Urbana-Champaign, Carle Illinois College of Medicine, Urbana, Illinois, United States

**Keywords:** Label-free imaging, optical microscopy, quantitative phase imaging, multiphoton imaging, multiharmonic imaging

## Abstract

**Significance:**

Imaging 3D *in vitro* kidney models is essential to understand kidney function and pathology. Label-free characterization of such specimens seeks to supplement existing imaging techniques and avoid the need for contrast agents that can disturb the native state of living samples. Conventional label-free optical imaging techniques are compatible with living samples but face challenges such as poor sectioning capability, fragmentary morphology, and lack of chemical-specific information.

**Aim:**

We aim to develop and demonstrate a correlative label-free imaging platform capable of simultaneously capturing morphological and chemical-specific information from 3D cultured kidney mesangial cells.

**Approach:**

We combined simultaneous label-free autofluorescence-multiharmonic (SLAM) microscopy and gradient light interference microscopy (GLIM) to extract both chemical-specific and morphological tomography of 3D cultured kidney mesangial cells. In this approach, SLAM provides a nonlinear imaging platform with a single excitation source to simultaneously acquire autofluorescence (FAD and NAD(P)H), second- and third-harmonic signals from the cells. Complementarily, GLIM acquires high-contrast quantitative phase information to quantify structural changes in samples with a thickness of up to 250  μm.

**Results:**

Our correlative imaging results demonstrate the ability to image and quantify both morphology and chemical-specific signals of kidney mesangial cells in 3D. The combination of GLIM and SLAM provides complementary information critical for understanding kidney function, including metabolism and matrix deposition under controlled physiological conditions.

**Conclusions:**

The proposed correlative imaging approach establishes a versatile and hassle-free platform for morpho-chemical cellular tomography, offering unique opportunities for studying the structure and function of 3D kidney models in their native state.

## 1 Introduction

The kidney is a complex and vital organ, and early detection of pathological changes is essential to prevent long-term complications in diseases such as glomerulonephritis and diabetic nephropathy. These disorders are strongly associated with metabolic dysregulation, where alterations in cellular metabolism often precede visible structural damage. Recent advances in 3D cell culture and tissue models enable the recreation of the kidney’s microenvironment in vitro, providing controlled systems to study cellular interactions, tissue organization, and disease mechanisms using optical microscopy. In this context, monitoring dynamic metabolic processes is critical for identifying early disease markers while quantitative morphological measurements are necessary to understand how these metabolic changes translate into spatial and structural alterations.

In diabetic nephropathy specifically, early mitochondrial dysfunction, oxidative stress, and impaired glucose metabolism drive downstream effects such as cytoskeletal remodeling, cell hypertrophy, altered adhesion, and disruption of the glomerular filtration barrier. Although these metabolic changes precede morphological damage, the mechanistic link between intracellular metabolic remodeling and subsequent structural alterations remains poorly understood, possibly due to the lack of correlative label-free methods capable of probing both metabolism and morphology simultaneously.

Label-free imaging seeks to enable direct observation of such biological samples in their natural state, potentially enabling real-time observations of biological processes with minimal perturbation. However, conventional bright-field microscopy suffers from low intrinsic contrast, which makes morphological imaging difficult, and a lack of chemical specificity, which hinders observations of biological processes. Emerging label-free imaging techniques seek to address some of these limitations—some in the realm of nonlinear optics, such as multiphoton autofluorescence and multiharmonic imaging,^1^ and others in the realm of linear processes, such as quantitative phase imaging (QPI),^2^ infrared,^3^ or Raman spectroscopic imaging,^1^^,^^4^^,^^5^ all of which have their unique and complementary contrast mechanisms.^6^ For example, QPI offers enhanced morphological imaging by utilizing an intrinsic optical contrast that encodes the thickness and local refractive index of the sample into the phase of the light passing through the sample.^5^ During the past few decades, a variety of QPI techniques have been developed to improve their performance in terms of resolution, axial sectioning, and spatial and temporal phase sensitivity.^7^^–^^9^ In general, it is challenging to choose the best existing QPI techniques due to multiple factors including resolution, acquisition speed, and particularly the suitability for specific applications.^9^^–^^11^ For example, only a few existing QPI techniques can provide morphological information for samples with thicknesses exceeding 100  μm.^12^^,^^13^ In addition, QPI lacks the chemical specificity required to distinguish features such as collagen, vesicles, and fibrosis, as well as to monitor physiologic processes that may not be manifest in morphologic changes.^5^^,^^7^^,^^10^ This limitation hinders the potential impact of QPI in biomedical applications and prevents its use as a complementary tool for developing more selective diagnostic biomarkers.

On the other hand, label-free imaging with multiphoton excitation of autofluorescence and harmonic generation can complement QPI in terms of chemical-specific imaging.^14^^,^^15^ Multiphoton microscopy (MPM) relies on multiphoton excitation, whereas multiharmonic microscopy (MHM) exploits the nonlinear scattering of the incident light at the sample to image deep within tissue.^16^ MPM encodes FAD and NAD(P)H coenzymes, which provide insight into the chemical environments of cells and tissues, such as oxidative and reductive states.^17^ For example, higher NADH generally indicates a more glycolytic state (less reliance on oxygen), often seen in rapidly dividing or hypoxic (low-oxygen) cells, such as cancer cells.^18^ By contrast, higher FAD indicates a more oxidative state, meaning that the cell is using oxygen to produce energy efficiently, as seen in healthy, well-oxygenated cells. In addition, MHM can visualize structural features such as actin, myosin, collagen fibers, extracellular vesicles, and water–lipid interfaces.^14^ As individual modalities, each provides an interesting but narrow window into cells and tissues. For example, the quality of the multiharmonic image depends on the spatial distribution of harmonophores, the phase of the fundamental beams, and phase matching, which may obfuscate quantitative interpretation of the resultant images.^19^ In addition, multiharmonic imaging provides contrast from boundaries and therefore encodes 2D morphology, but cannot indicate the optical thickness aspect of morphology.

Interestingly, the picture becomes more complete by performing correlative imaging using more than one imaging technique. Some strides have been made to support correlative multimodal label-free imaging, such as the simultaneous label-free autofluorescence-multiharmonic (SLAM^18^) system and multimodal hyperspectral imaging systems.^20^ However, integration with QPI systems has been quite challenging so far due to (a) use of the optical components from a different spectral window for both illumination and collection and differences in optical system design, (b) limited depth of penetration of QPI,^5^ and (c) difficulty in correlating volumetric morphological information imaged in QPI with single plane information encoded in nonlinear label-free imaging modalities. Due to these challenges, the complementarity of QPI over the other techniques has not been significantly investigated. Although gradient light interference microscopy (GLIM)^13^ solves problem (b), we demonstrate a solution to problem (c) and therefore illustrate both the correlative and complementary aspects of combining QPI with multiphoton autofluorescence and multiharmonic systems.

In response to these challenges, we introduce a multimodal correlative label-free imaging approach to reveal both morphological and chemical-specific information, and to measure the 3D nanoscale changes and cellular trafficking in fixed kidney mesangial primary cells. We used two different optical microscopes to perform the correlative imaging of fixed kidney cells. Correlative imaging^21^ with QPI and label-free chemical imaging techniques has been demonstrated previously as well.^21^^,^^22^ For example, Arianna et al.^23^ showed label-free morpho-molecular imaging of cancer cells using Raman spectroscopy and phase tomography. Rishikesh et al.^24^ employed diffraction phase microscopy and Raman imaging for morpho-molecular imaging of live cells. However, most of these techniques either provide 2D images or have limited depth of penetration.^25^ By contrast, the present work utilizes the nanoscale sensitivity of GLIM^13^ to extract the morphological information of 3D mesangial cells. In addition, SLAM^18^ microscopy supports the simultaneous quantification of morphological, metabolic, and structural changes in samples up to 250  μm thickness. Therefore, correlative imaging using GLIM and SLAM allows nanometric morphological changes along the axial direction (with GLIM) and chemical-specific imaging (with SLAM) at each plane of the sample. In this work, we demonstrate the capability of the system to extract morpho-chemical tomography of a 3D kidney organotype and investigate cellular appendages such as filopodia. The 3D morphology and function of mesangial cells are investigated using GLIM and SLAM to quantify FAD, NAD(P)H, collagen, and optical heterogeneity to better understand their functions, including metabolism, matrix deposition, and roles in kidney pathology. Correlating SLAM (molecular/metabolic information) with GLIM (quantitative structural information) helps to track intracellular processes related to structural remodeling in a label-free manner, which cannot be achieved using either modality alone. Thus, the correlative approach offers a combination of functional and quantitative information about the cells with large depth of penetration, providing an imaging platform with potential for high-throughput chemical and morphological imaging for specific biological applications.

## 2 Methods

### 2.1 Experimental Design and System Configuration

**Fig. 1:**
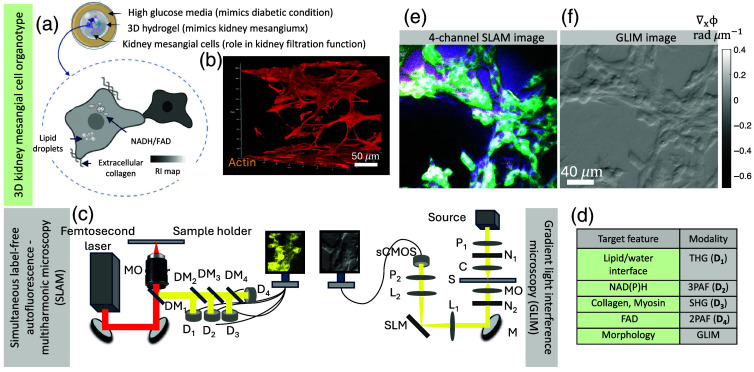
Illustration of the study design. (a) Shows the configuration of the 3D organotypic of kidney mesangial microenvironment that dictates the renal filtration function. (b) 3D image of the model when the sample is fixed with actin labeled. The intracellular, extracellular, and cell–cell interactions determine the mesangial function and can be chemically imaged with (c) correlative SLAM (left) and GLIM (right) imaging setup. (d) Outlines the different aspects of the mesangial functions that can be targeted with the inverted imaging setup. (e): 4-channel SLAM image and (f) GLIM image. D: detector, DM: dichroic mirror, MO: microscope objective, $P_{1-2}$: polarizer, $N_{1-2}$: DIC prism, C: condenser lens, S: sample, M: mirror, $L_{1-2}$: lens.

Figure 1 shows a representative 3D cultured model of kidney mesangial cells that are responsible for crucial functions in the blood filtration process. High sugar concentrations during diabetic nephropathy affect the function of the mesangial cells.^26^ The disease is manifested due to factors such as glucose levels, blood pressure, and inflammation, which result in stiffening of the matrix and thereby lead to compromised function. Figure 1(a) illustrates the structural and functional components that can be exploited for understanding the functionality of mesangial cells. Figure 1(b) shows the 3D image of the model labeled actin (a structural protein). Figure 1(c) shows the schematic of the multimodal label-free imaging approach used to visualize both morphology and chemical-specific information and to measure inter-cellular trafficking in cultured kidney mesangial cells. SLAM^18^ and GLIM^13^ systems shown in Fig. 1(c) are utilized to extract metabolic/structural changes and nanoscale morphology of the sample, respectively. Here, two different custom-built SLAM and GLIM systems are used to perform morpho-chemical tomography. Note that both modalities can provide morphological and metabolic changes in samples with a thickness of up to 250  μm.^13^^,^^18^ Therefore, correlative imaging using GLIM and SLAM enables high-resolution morphological imaging along the axial direction (with GLIM) and chemical-specific imaging (with SLAM) at each plane of the sample.

Co-registration of images between the two modalities is performed to approximately match the field of view. A gridded glass-bottom Petri dish was used, where the etched grid served as fiducial markers to provide common spatial references visible in both imaging modalities. The sample was first imaged with GLIM (20×, 0.45 NA), where the fiducial markers were clearly identifiable. Subsequently, the same field of view was located in the SLAM system by manually translating and rotating the motorized stage until the identical fiducial markers were observed.

Because GLIM and SLAM use objectives with different numerical apertures (0.4 NA versus 1.05 NA), the acquired images exhibit different spatial resolutions and pixel sizes. To address this, both datasets were imported into MATLAB. The SLAM images were resampled/interpolated to match the GLIM pixel grid, after which an initial rigid registration (translation and rotation) was performed using the fiducial markers. This was followed by a fine alignment step based on intensity-based cross-correlation to minimize residual mismatch.

SLAM (20×, 1.05 NA) microscopy captured NAD(P)H, FAD autofluorescence, second harmonic, and third harmonic signals. A single excitation band from 1080 to 1140 nm is used to achieve simultaneous molecular contrast visualization in four detection channels. Near-transform-limited pulses with broader bandwidth enhance contrast, enabling a clearer interpretation of intercellular dynamics. SLAM combines 2-photon autofluorescence (2PAF), 3-photon autofluorescence (3PAF), second harmonic generation (SHG), and third harmonic generation (THG) imaging to extract FAD, NAD(P)H, collagen, and lipid–water interfaces from the kidney mesangial cells, respectively. In the SLAM system, a 20×, 1.05 NA lens is used for the imaging. The system used near-transform-limited excitation pulses with bandwidth of 60 nm, and a pulse duration of 35 fs at a low pulse repetition rate (10 MHz), resulting in higher peak power compared with standard pulses with an average power of 14 mW at the sample surface. The sectioning depth and spatial resolution of the system are 200  μm and 500 nm, respectively. The acquisition speed (temporal resolution) of the system is ∼18  s per 900×900  pixel image. Further, different dichroic mirrors and optical filters are used in the detection system to collect spectrally resolved multiphoton and multiharmonic signals by photomultipliers [Fig. 1(d)]. More details of the SLAM system can be found the literature.^18^

In addition, GLIM (20×, 0.45 NA) is used to extract optical phase tomography of the cells.^27^ GLIM combines differential interference contrast (DIC) with a phase-shifting unit to extract the gradient phase at each layer of the sample. Selective interference of DIC and phase shifting suppresses unwanted scattering in multilayer samples and therefore helps produce tomography images of both thin (300 nm) and thick samples of up to 250  μm.^13^ The GLIM system provides a quantitative phase gradient map of the sample at the resolution of the objective lens. In this work, we used a 20×, 0.45 NA lens to acquire the GLIM image with a step size of Δz=0.5  μm. The lateral and axial resolutions of the system are 700 and 780 nm, respectively. The GLIM system operates at 10 phase images per second. Further details of the GLIM system can be found in the literature.^13^

### 2.2 Sample Preparation

A total of 12 samples with varying stiffness were prepared for the study. To prepare the samples, mesangial cells were commercially procured and grown in Dulbecco’s Modified Eagle Medium (DMEM) with high glucose (4.5  g/L) supplemented with 20% fetal bovine serum (FBS) and 1% penicillin–streptomycin antibiotic. For the 3D cell culture, we used in-house prepared gelatin methacryloyl (GelMA). A total of 2.5% (soft) and 5% (normal) (w/v) GelMA foam was dissolved in 1× phosphate buffered saline solution (PBS) and lithium phenyl-2,4,6-trimethylbenzoylphosphinate (LAP) was used as the photoinitiator. LAP was mixed with GelMA at a concentration of 5  mg/mL to make the precursor solution. GelMA was then cast on 35 mm glass bottom dishes and crosslinked with UV lamp exposure. Mesangial cells were cultured in soft and normal stiffness GelMA hydrogels and allowed to grow for 96 h.

For mitochondria labeling, the cells were first fixed with 4% paraformaldehyde in PBS for 20 min. The cells were permeabilized in 0.3% Triton X-100 permeabilization buffer with 5% goat serum. Rabbit monoclonal Anti-TOMM20 antibody—the mitochondrial marker (AB186735, Abcam)—was used as the primary antibody, which was incubated with the sample overnight at 4°C. For fluorescence imaging, a goat anti-rabbit secondary antibody tagged with Alexa Fluor 647 (A21245, Invitrogen) was incubated for 45 min before washing. Imaging was done using a laser scanning confocal microscope (Zeiss LSM-800) with a 40× (0.8 NA) objective lens. Some samples were labeled with actin for visualizing the distribution of cells in the 3D hydrogel. The fixed and permeabilized samples were incubated with Phalloidin Atto-647 for 45 min, then washed and imaged using a confocal microscope.

Although both GLIM and SLAM are inherently compatible with live-cell imaging, in the present study, the two modalities are not integrated into a single platform but are implemented on two separate microscope systems. Therefore, imaging was performed sequentially rather than simultaneously. In preliminary experiments with live cells, we observed that cell motion, morphological changes, and stage repositioning between instruments made it difficult to reliably image the identical field of view across both modalities, which compromised accurate co-registration. To ensure spatial correspondence and reproducibility between the SLAM and GLIM datasets, the cells were fixed. Fixation immobilized the cells and preserved their morphology, enabling consistent relocation of the same region of interest (ROI) and reliable multimodal alignment.

## 3 Results and Discussion

**Fig. 2:**
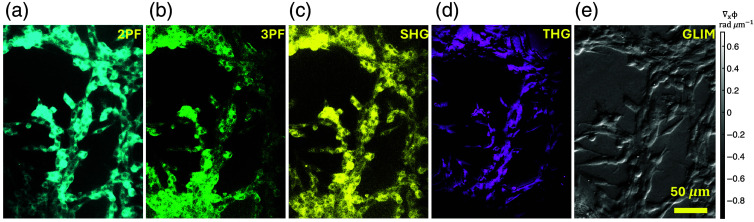
Comparative visualization of the mesangial organotype in the (a)–(d) SLAM and (e) GLIM imaging systems for the primary kidney mesangial cells. The GLIM images provide the gradient phase values of the cells which aid in identifying the content of the cells and their spatial distribution. The corresponding field of the SLAM images shows the metabolic and chemical profile of the cells including NAD(P)H, FAD, lipids, and actomyosin. Cyan: 2PF, green: 3PF, yellow: SHG, magenta: THG, gray: GLIM image.

Using an average power of 14 mW at the sample surface, SLAM images were acquired from a 3D kidney mesangial cell culture in soft and normal matrices *in vitro*. A total of 12 replicate samples of 3D hydrogels with kidney mesangial cells were visualized under the SLAM system. Multiple images were acquired from various ROI (∼25 FOV per sample). Each image was taken over 18 s. Different portions of these cells are identifiable based on their distinct features from the four-channel-based optical signatures, as shown in Fig. 2. The raw four-channel image was loaded into Fiji, an open-source platform for image analysis, to apply pseudo-color maps and merge the contrast. The pseudo-color maps represent the intensity of the image. The same color maps were used consistently throughout the study, namely cyan for 2PF, green for 3PF, yellow for SHG, and magenta for THG. This specific color scheme was chosen to improve the contrast between the cellular and extracellular components of interest. Cells with localized 2PAF (cyan, FAD for nonfixed cells) and 3PAF (green, NAD(P)H for nonfixed cells) signals show cytoplasm, cell shapes, sizes, and nuclei, as shown in Figs. 2(a) and 2(b).

In the mesangial cells that demonstrate a high metabolic state under diabetic conditions, the spatial distribution of 2PAF and 3PAF provides a window to measuring NAD(P)H and FAD. However, as we used fixed samples to perform correlative imaging, this affected the optical redox ratio (ORR). The 2PF (used for FAD) and 3PF (used for NAD(P)H) signals, which are essential components of ORR measurements, were altered after fixation. Fixation introduces fluorescence from the fixative itself, resulting in 2PF reflecting a mixed signal of FAD + fixative and 3PF a combination of NAD(P)H + fixative. Therefore, the accurate ORR measurements using FAD and NAD(P)H are possible only with fresh, nonfixed cells.

In addition to the shape and size of cells, our imaging of kidney mesangial cells in normal matrices *in vitro* [Figs. 2(c) and 2(d)] indicates overall structural changes, changes in the metabolic state, and cellular production of matrix proteins such as cytoskeletal structures and lipid bodies. As mesangial cells regulate glomerular blood flow through their contractile actomyosin machinery, SHG [Fig. 2(c)] can be used to detect changes in cytoskeletal organization and contractility, enabling the monitoring of early functional alterations associated with glomerular dysfunction in live cells. In addition, THG [Fig. 2(d)] signals are primarily associated with the third order susceptibility and provide contrast from vesicles and boundaries of subcellular structures of the mesangium.

It is worth noting that most cells under normal conditions—save cardiomyocytes, fibroblasts, and, as we have shown in this study, the mesangial organotype—produce negligible SHG. In addition, our SLAM system relies on carefully chosen filters and dichroic mirrors to diminish or even completely remove crosstalk. Therefore, the substantial contrast we observe in the SHG channel [Fig. 2(c)] is more consistent with the potential of actomyosin to drive SHG signals than with bleed-through from the 2PAF/3PAF channels or contributions from intrinsic actomyosin autofluorescence centered ∼300 to 360 nm. Finally, no cross-channel subtraction or unmixing was applied.

Further, SLAM findings are correlated with GLIM to understand the relationships among metabolism, fibrosis, and morphology, which are crucial for studying kidney disease *in vitro*. We manually registered an approximately similar field of view between SLAM and GLIM using MATLAB.^28^ Specifically, the GLIM image was cropped and rotated in MATLAB to approximate the field of view of the SLAM image. Due to the distinct morphological and chemically specific regions highlighted by the GLIM and SLAM systems, the correlation analysis shows poor alignment between the registered images. Nonetheless, using MATLAB, it is possible to crop the GLIM image to approximate a similar field of view and align it with SLAM images. GLIM has a unique capability of suppressing the multiple scattering from the defocused layer of the thick samples. Partial coherence illumination in GLIM, selective interference in DIC, and four-phase shifting help reject multiple scattering, thus providing high-contrast imaging for thick samples, which is unusual in other conventional phase imaging techniques. Figure 2(e) shows the GLIM image of kidney mesangial primary cells. Removing multiple scattering using GLIM helps to visualize intercellular details of complex 3D cellular systems. Figure 2(e) represents the derivative of the phase at each point of a sliced portion of mesangial cells, which demonstrates the ability of GLIM to visualize through cellular layers and is well suited for applications such as 3D tissue imaging. The ability to recover overall morphology and chemical-specific signals in thick samples hints at the potential of integrating these techniques in future use in *in vivo* applications.

**Fig. 3:**
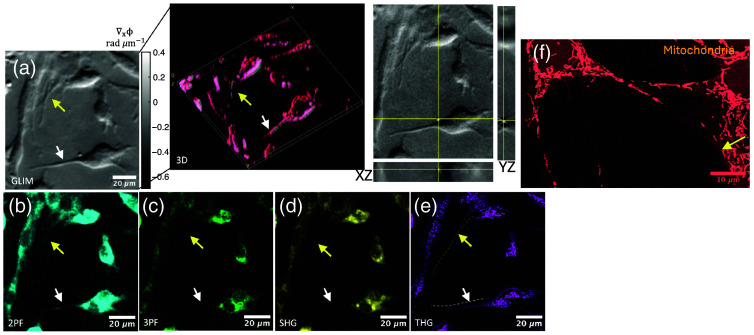
Figure illustrates cell–cell interaction among mesangial cells and the relevance of the SLAM-GLIM correlative imaging. (a) GLIM image and its 3D revisualization show the filopodia that function in coordinating activities in the mesangium. Two distinct filopodia are indicated by yellow and white arrows. (b) In 2PAF, the white-arrowed filopodia are very prominent, whereas the yellow-arrowed filopodia are sparingly visible. (c) In 3PAF and (d) SHG, the white-arrowed filopodia are sparingly visible but the yellow-arrowed filopodia are not visible at all. (e) The filopodia are visible in the THG image. (f) A fixed and labeled mitochondria region (same sample, different ROI for representation) shows white-arrowed filopodia with several mitochondria being transferred, whereas yellow-arrowed filopodia are mostly empty and only one mitochondrion at its distal end.

To demonstrate the relevance of the correlative imaging, we investigated the cellular appendages or filopodia (Fig. 3). In cells, these appendages can range from sub-micron to sub-diffraction sizes. Tunneling nanotubes (TNTs) are such filopodial extensions that are responsible for exchanging key organelles such as mitochondria, vesicles, and nutrients between cells.^29^ The exchange process is key for the physiological functioning of the cells and their alteration can be indicative of pathology. In kidney glomerular cells, they are responsible for proper renal filtration function.^30^ Such structures are replete with mitochondria, which means the metabolic activity of NAD(P)H and FAD can be accessed. Further, the region will have actomyosin complexes for providing structure, as well as lipids from the cell membrane and lipid vesicles as transporters of various proteins. Therefore, such a region has the potential for being investigated using multiphoton and multiharmonic imaging. In Fig. 3(a), we observe two different types of filopodia marked with white and yellow, showing distinct phase gradients. This implies the dry mass of the white-arrowed filopodia is higher.^13^^,^^31^ Although 2-photon imaging showed the highest signal for the white-arrowed filopodia, the yellow-arrowed filopodia were only sparingly visible [Fig. 3(b)]. As 2-photon fluorescence captures FAD, the filopodia likely have a high density of mitochondria being transferred within. Figure 3(f) shows a filopodium with high and low mitochondria content as it appears when labeled and imaged. In the 3-photon modality for imaging the NAD(P)H [Fig. 3(c)], although the filopodia are sparingly visible, the white arrowed one is brighter. In SHG [Fig. 3(d)], we again observe very little signal from either filopodium, with the denser filopodium (white arrow) showing a slightly higher signal. This is expected, as the only source of noncentrosymmetric structure is myosin; thus, the density of myosin determines the brightness. The samples were cultured for less than a week and hence substantial collagen was not deposited by the cells to be visualized by SHG. In the THG image [Fig. 3(e)], we interestingly observe that both filopodia are equally visible with nearly similar intensity. This is expected as both structures have only a single lipid bilayer around the appendage, yielding equal signal strength.

As different optical systems and magnifications are used to acquire both SLAM and GLIM images, fiducial markers and post processing using MATLAB were used to locate the same ROI. However, subpixel matching of GLIM and SLAM images is challenging due to different resolution, magnification, sample rotation, and mechanical vibrations of the different imaging systems. Nonetheless, the 2PAF, 3PAF, SHG, and THG images from the common ROI complement the GLIM image to extract metabolic and structural properties of the sample. Unlike marker-based microscopy techniques, the intensity at four different channels in SLAM microscopy provides 3D structural and functional imaging of cellular and subcellular structures without the need for exogenous contrast. Although correlative GLIM and SLAM imaging is performed to extract both linear and nonlinear susceptibilities of the sample, our future aim will be to integrate these modalities to provide a common imaging platform for *in vivo* live cell imaging.

## 4 Conclusion and Future Scope

Here, we present a proof-of-principle that complementary label-free techniques such as GLIM and SLAM can serve as valuable tools for extracting morpho-chemical tomography of biological samples. The primary motivation of this work is to underscore the importance of correlative morphological and chemical imaging in identifying insights from 3D biological organotypic models. We present the importance of correlative morphological and chemical imaging to identify such clues from 3D biological organotypic models of the kidney mesangium. The kidney is a sensitive organ, and it is crucial to detect early pathological changes to avoid irreversible damage. Mesangium is a key microenvironment in the body that resides upstream of the disease development pathway. Thus, imaging such a structure will be crucial to identify early disease markers. We demonstrated that correlative label-free imaging methods can be useful to extract morphological and metabolic signatures from the 3D cultured cells.

In the context of diabetic nephropathy, subtle morphological and biophysical changes often precede clear structural alterations. GLIM enables sensitive, label-free quantification of these changes, allowing us to assess variations in cellular mass distribution and intracellular organization that are not directly accessible through SLAM imaging. Therefore, the co-registration of GLIM and SLAM provides complementary information: SLAM offers molecular and structural contrast, whereas GLIM adds quantitative morphological insight. Although more structural biological studies need to be planned, for example, through renal biopsies to address individual diseases such as diabetic nephropathy, where diagnosis is very challenging because it silently affects the organ. Currently, the SLAM and GLIM setups are housed in different microscopes in this study. However, the study confirms the need for co-localized imaging, which is crucial for identifying live cells and their sub-cellular organelles and dynamics at small scales, ranging from sub-micron to the optical diffraction-limited resolution. As many chemical changes occur at high resolution, balancing resolution and signal-to-noise ratio for 3D samples is important. We also acknowledge the need for fast imaging as the cell–cell organelle transfers occur at significantly faster time scales.^30^

## Data Availability

The dataset will be made available by the corresponding author upon reasonable request.
